# Fibroblasts Cultured on Nanowires Exhibit Low Motility, Impaired Cell Division, and DNA Damage

**DOI:** 10.1002/smll.201300644

**Published:** 2013-06-27

**Authors:** Henrik Persson, Carsten Købler, Kristian Mølhave, Lars Samuelson, Jonas O Tegenfeldt, Stina Oredsson, Christelle N Prinz

**Affiliations:** The Nanometer Structure Consortium, Lund University, Box 118, 22100 LundSweden; Center for Electron Nanoscopy, Technical University of Denmark, Ørsteds Plads 345E, 2800 Kongens LyngbyDenmark; Department of Micro- and Nanotechnology, Technical University of Denmark, Ørsteds Plads 345E, 2800 Kongens LyngbyDenmark; Department of Biology, Lund University, Sölvegatan 37, 223 62 LundSweden; Neuronano Research Center, Lund University, Sölvegatan 19, 221 84 LundSweden

## Abstract

Nanowires are commonly used as tools for interfacing living cells, acting as biomolecule-delivery vectors or electrodes. It is generally assumed that the small size of the nanowires ensures a minimal cellular perturbation, yet the effects of nanowires on cell migration and proliferation remain largely unknown. Fibroblast behaviour on vertical nanowire arrays is investigated, and it is shown that cell motility and proliferation rate are reduced on nanowires. Fibroblasts cultured on long nanowires exhibit failed cell division, DNA damage, increased ROS content and respiration. Using focused ion beam milling and scanning electron microscopy, highly curved but intact nuclear membranes are observed, showing no direct contact between the nanowires and the DNA. The nanowires possibly induce cellular stress and high respiration rates, which trigger the formation of ROS, which in turn results in DNA damage. These results are important guidelines to the design and interpretation of experiments involving nanowire-based transfection and electrical characterization of living cells.

## 1. Introduction

During the past decade, nanotechnology has generated an increased interest for its anticipated applications in medical and biological research. Vertical semiconductor nanowire arrays is one example of new structures currently finding their way into biological applications. Their small dimensions and high aspect ratio (typically diameters below 100 nm and lengths ranging from 1–10 μm) make the nanowires ideally suited for interfacing living cells^1^, ^2^ for applications including cellular force measurements,^3^ electrical recordings,^4^–^6^ physical transfection^7^–^12^ and cell guidance.^13^–^14^

Most current studies focus on how nanowires can be used to manipulate cells while few studies investigate how cells are affected by these structures. Specifically, for experiments where nanowires are used as a tool to manipulate or characterize the cells, it must be ensured that (a) any influence on cell behaviour is minimized and (b) that the effects of the nanowires are well understood. Some studies simply assessed the cell viability using respiratory assays or membrane impermeable dyes,^1^, ^7^, ^15^ while others have examined the expression of a small selection of housekeeping genes.^11^, ^15^ Other groups have also investigated the cell-nanowire conformation using fluorescence microscopy, transmission electron microscopy (TEM), or focused ion beam (FIB) milling combined with scanning electron microscopy (SEM).^16^–^17^–^18^–^19^ Among the many cell properties that are likely to be influenced by substrate topography, cell migration seems to be of particular interest, since it has been shown to vary greatly between different nano-topographies, for which no cell proliferation changes were observed.^20^ Although many of the nanowire array application studies use immortalized cells that often are highly motile and dividing, there is limited data available concerning the effects of nanowire arrays on the dynamics of the cells, such as cell migration or cell division.

Here, we have performed live cell imaging using phase holographic microscopy to investigate cell motility and cell division on nanowire arrays. We have used immortalized mouse fibroblasts (L929) and vertical gallium phosphide (GaP) nanowires with three different lengths (1.5 μm, 3.8 μm, and 6.7 μm). We show that cell motility and cell division are greatly reduced by the nanowires. The long nanowires impede the cell division, leading to the formation of multinuclear cells. Fibroblasts cultured on nanowires have a higher content of reactive oxygen species (ROS), higher respiration rate and show signs of DNA damage compared to control cells. Using FIB milling and SEM imaging, we show that there is no direct nanowire–DNA contact and we therefore hypothesize that the DNA damage is caused by the high ROS cell content, which in turn may be caused by the observed high respiration rate.

We expect our findings to influence the design process of nanowire-based platforms for live cell studies as they demonstrate a surprisingly strong influence of the nanowires on cell behaviour compared to previous studies. In order to minimize cell perturbation, our results suggest that the nanowire length should be minimized and the cell–nanowire interaction time should be kept short to avoid any interference with the cell division process.

## 2. Results

**Figure 1:**
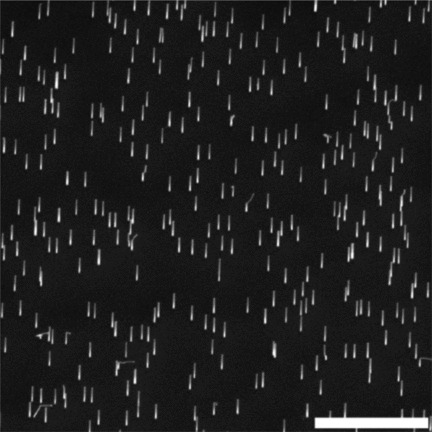
Scanning electron micrograph of a vertical GaP nanowire substrate. The nanowires are 3.8 μm long. Stage tilt 30°. Scale bar 5 μm.

We cultured L929 fibroblasts on vertical GaP nanowires, randomly positioned on the substrate, with an average density of 1 nanowire per μm^2^ (**Figure** **1**).

### 2.1. Reduced Cell Motility on Long Nanowires

**Figure 2:**
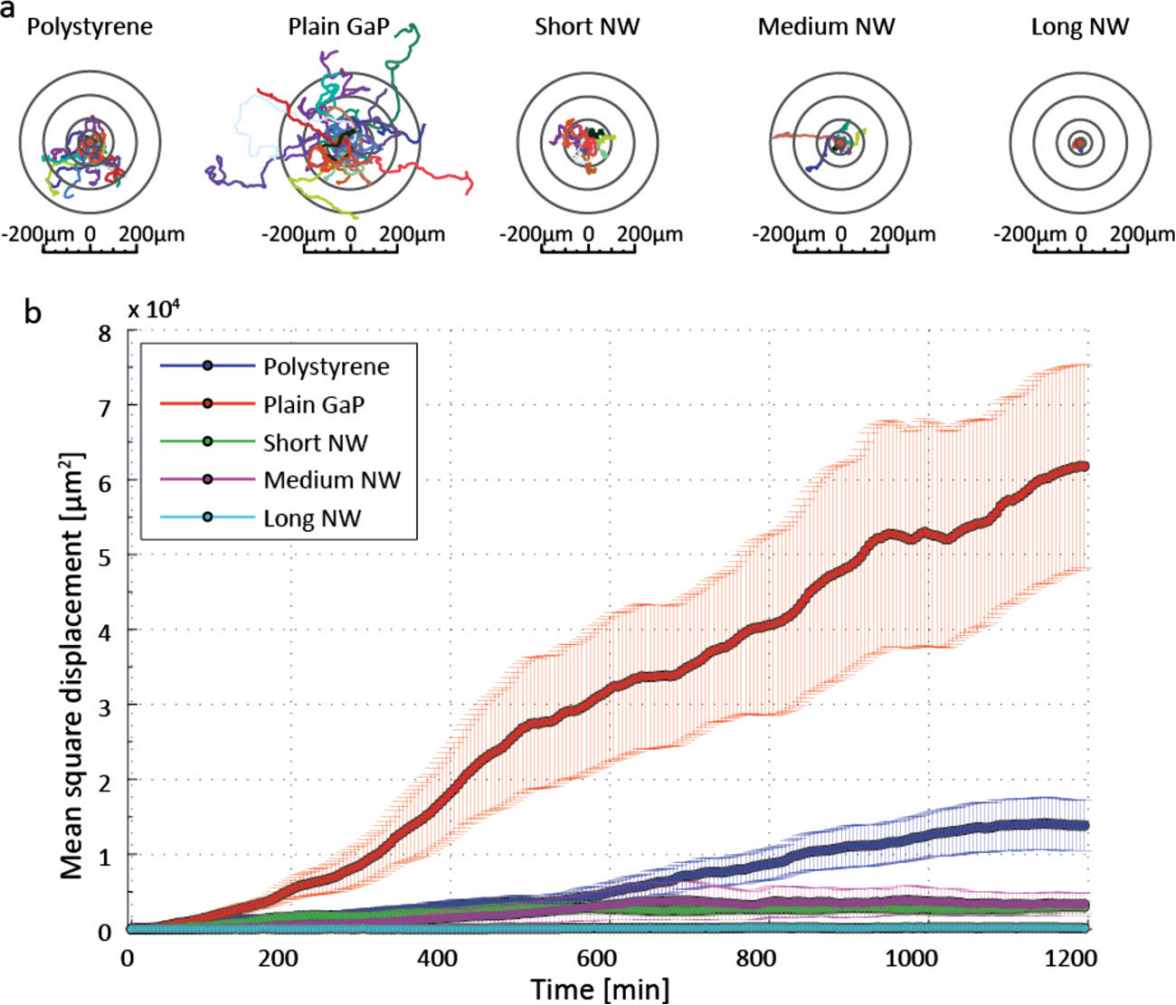
Fibroblast motility analysis. (a) Twenty individual cells tracked for 20 hours on each substrate: polystyrene, plain GaP, short, medium and long nanowires (NW). (b) Mean square displacement of the 20 cells plotted as a function of time. Mean value ± S.E.M.

Using phase holographic microscopy, we tracked the fibroblast displacement on short (1.5 μm), medium (3.8 μm) and long (6.7 μm) nanowire substrates, as well as on control substrates (Supporting Movies S1–S6). Our results show that the cell motility is greatly reduced on the nanowires compared to control polystyrene (PS) and planar GaP substrates as shown in the plot of mean square displacement (MSD) over time in **Figure** **2**. Fibroblasts cultured on long nanowires are completely non-motile. Notably, cells are significantly more motile on planar GaP compared to PS substrates, which could be attributed to poor cell adhesion on the very smooth GaP substrate.^21^

### 2.2. Nanowires Reduce Rate of Cell Proliferation

**Figure 3:**
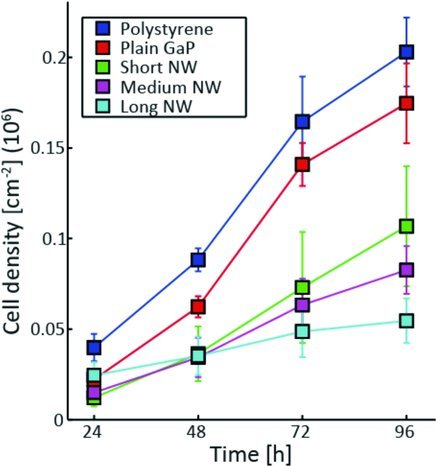
Cell proliferation rate. Growth curves on the five different types of substrates as indicated in the legend (nanowires are noted NW). The cell density was determined using phase holographic microscopy. Mean value ±S.E.M.

Since cells are immobile during cell division, we investigated whether the measured decrease in motility could be explained by an increase in cell proliferation. We determined the cell density on the different substrates and found that the cell proliferation rate is similar for both PS and GaP control substrates, but significantly lower on the nanowire substrates, where it decreases with increasing nanowire length (**Figure** **3**). This observation rules out any possible link between the observed cell motility and proliferation behaviors.

In order to investigate whether fibroblasts cultured on nanowires could reach confluency, a long-term growth experiment was carried out. Cells were cultured for 360 h (15 days) in polystyrene culture flasks, as well as on short and long nanowire substrates The resulting growth curves (Supporting Figure S2) show that fibroblasts cultured on long nanowires do not reach confluency but rather decrease in number after 96 h. In contrast, the cells on short nanowires keep increasing in number throughout the experiment and reach confluency and the cells on the polystyrene substrate grow in multiple layers.

### 2.3. Long Nanowires Interfere with Division

**Figure 4:**
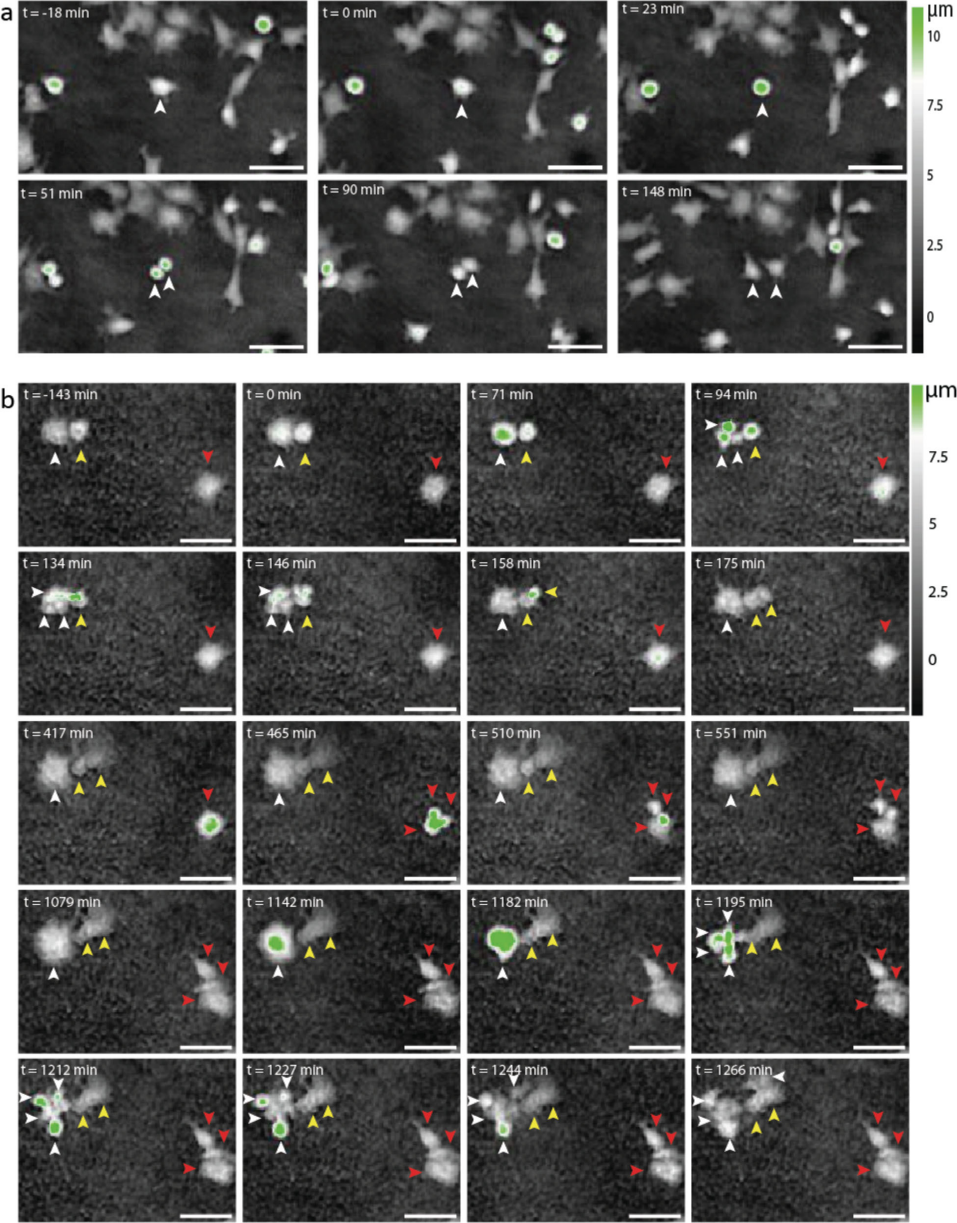
Representative phase holographic time-lapse images of dividing L929 fibroblasts. (a): cell behavior on polystyrene control substrates. These frames show a cell (white arrow) dividing by rolling up and splitting in two distinct cells. Time 0 was chosen as the time when the cell protrusions started to retract and the cell started to roll-up. The total division time was about 90 min. (b): Anomalies in cell division on long nanowires. The cell marked with a white arrow attempted to divide into three daughter cells (t = 0 through t = 158 min). Cell division was aborted and the cell spread on the surface as one large cell (t = 175 min through t = 1079 min). It eventually divided into 4 daughter cells (t = 1142 min through t = 1266 min). The cell highlighted with a yellow arrow, divided successfully with the two daughter cells spreading on the surface after the division. The cell marked with a red arrow divided into three daughter cells (t = 417 through t = 551 min). Scale bars 50 μm.

In order to investigate why the rate of cell proliferation was reduced on nanowires compared to controls, we used time-lapse movies to study cell-division events more closely on the different substrates. Selected frames from time-lapse movies of cells cultured on long nanowires and flat polystyrene are shown in **Figure** **4**. On the polystyrene substrate (Figure 4a and also Supporting Movie S1), cells readily roll-up, divide, and the two daughter cells separate and spread again on the surface (white arrows in Figure 4a), as expected. We observed a similar behavior of cells cultured on planar GaP and short nanowires (Supporting Movies S2, S3). However, in many cases on long nanowires (Figure 4b), cell division is initiated with the cell adopting a rolled-up morphology, but the process is prolonged and often aborted and the cell spreads-out on the substrate, becoming a very large cell (white arrow in Figure 4b). Note that 984 min after the first attempt to divide (at t = 1142 min), the cell highlighted with a white arrow in Figure 4b divided into four daughter cells. Cell divisions resulting in several daughter cells were seen frequently on long nanowire substrates (also indicated by the red arrows in Figure 4b). Note that apart from during cell division, the cells in these frames did not move at all over a period of almost 24 h. Similar anomalies in cell division were observed when cells were grown on medium-nanowire substrates, but to a lesser degree (Supporting Movie S4).

### 2.4. Aborted Division Leads to Multinuclear Cells

**Figure 5:**
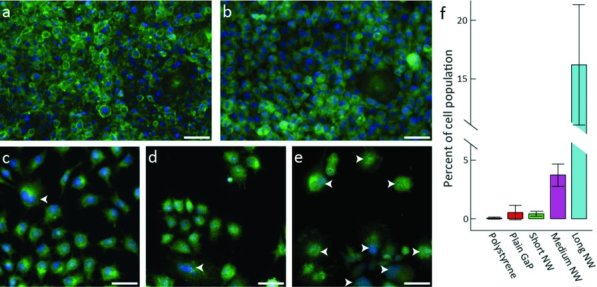
Occurrence of multinuclear cells on nanowire substrates and controls. Fluorescence microscopy images of cells cultured for 96 h on (a) polystyrene substrate, (b) planar GaP substrate, (c) short nanowire substrate, (d) medium nanowire substrate and (e) long nanowire substrate. The actin filaments were labeled using FITC-conjugated phalloidin (green) and cell nuclei were stained with bisbenzimide (blue). Multinuclear cells are highlighted by arrows. Scale bars 50 μm. (f): Proportion of cells with more than one nucleus (72 h culture). Error bars show the standard deviation.

To further study the aborted cell divisions observed on the long nanowires, we used fluorescence labeling to investigate the morphology of the cells cultured on the different substrates. **Figure** **5** shows fluorescence microscopy images of fibroblasts cultured for 96 h on planar control substrates (a,b) and on nanowire substrates (c–e). The cells were confluent on both polystyrene (Figure 5a) and planar GaP (Figure 5b) substrates, while the lower cell number on the nanowire substrates reflected the lower rate of cell proliferation shown in the growth curves (Figure 3). The cells cultured on the control substrates appeared homogeneous in size while the cells on nanowire substrates showed a greater variability in size and shape. Large multinuclear cells on the medium and long nanowire substrates could be seen, which are univocally the results of aborted cell divisions. Here we chose to use the term multinuclear since it seems to describe the majority of abnormal cells found on the substrates. However, it should be noted that this terminology also includes possible polyploid large nucleus cells. Hence the abnormal cells are characterized by an increase in DNA content, resulting from either uncompleted cytokinesis with completed karyokinesis (multinuclei) or incomplete cyto- and karyokinesis (polyploid large nucleus). The proportion of multinuclear cells is large on the long nanowire substrate (16 ± 5%) compared to the polystyrene substrate (0.1 ± 0.1%), the short (0.4 ± 0.2%) and the medium nanowire substrates (3.7 ± 1.0%) (Figure 5f and also Supporting Table S1). Cells cultured for 360 h show a similar trend, with very few multinuclear cells on short nanowires (2 ± 1.5%) and many on the long nanowires (31 ± 8%), (Supporting Figure S3, S4, and Table S2).

### 2.5. DNA Double Strand Breaks in Fibroblasts Cultured on Nanowires

**Figure 6:**
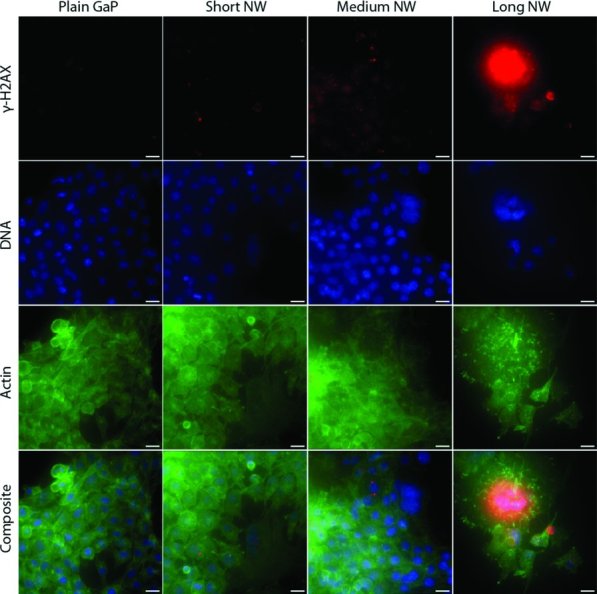
DNA double strand breaks investigated by γ-H2AX detection. Mouse L929 fibroblasts were cultured for 96 h on nanowire and control substrates. After fixation, primary antibodies against γ-H2AX and secondary Alexa Fluor-594 conjugated antibodies were used to detect γ-H2AX (red). Actin was labeled with FITC-labeled phalloidin (green) and DNA was stained using bisbenzimide (blue). Fewer and larger (multinuclear) cells could be seen on the long nanowire substrate (right panel), where most cell nuclei stained positive for γ-H2AX. Scale bars 20 μm.

In order to investigate whether nanowires induce any DNA damage, the presence of γ-H2AX was investigated using immunofluorescence microscopy. Double-stranded DNA damage induces phosphorylation of the histone H2AX, thereby forming γ-H2AX.^22^ The cells were cultured on plain GaP and nanowire substrates for 96 h and labeled for γ-H2AX. No γ-H2AX positive cells were found in cultures on plain GaP (**Figure** **6**, left panels). When cells were cultured on medium and short nanowires, there were a few γ-H2AX positive cells (Figure 6, middle panels). On long nanowires, most cell nuclei (blue) are γ-H2AX positive (red) (Figure 6, right panel). These results indicate that nanowires induce DNA damage. We found γ-H2AX positive nuclei in both single and multinuclear cells.

### 2.6. Fluorescence Microscopy Shows Indications of Holes in the Nuclei

It is known that DNA damage can either be caused by a direct nanowire-DNA contact, or by other indirect mechanisms.^23^ Using fluorescence microscopy, we found indications that cell nuclei were penetrated or indented by long nanowires. Close-up fluorescence images of cells on long nanowires show dark dots in the nuclei, co-localized with bright actin dots (Supporting Figure S5). We have recently reported dark dots in nuclei with DNA staining, co-localized with bright actin dots in fibroblasts cultured on ordered arrays of hollow nanowires.^23^ Each spot could then clearly be linked to the presence of a nanowire at that precise location, which could be suggesting that the nucleus was either pierced or indented by the nanowires.

### 2.7. Cell Nucleus Membrane Folds to Avoid the Nanowires

**Figure 7:**
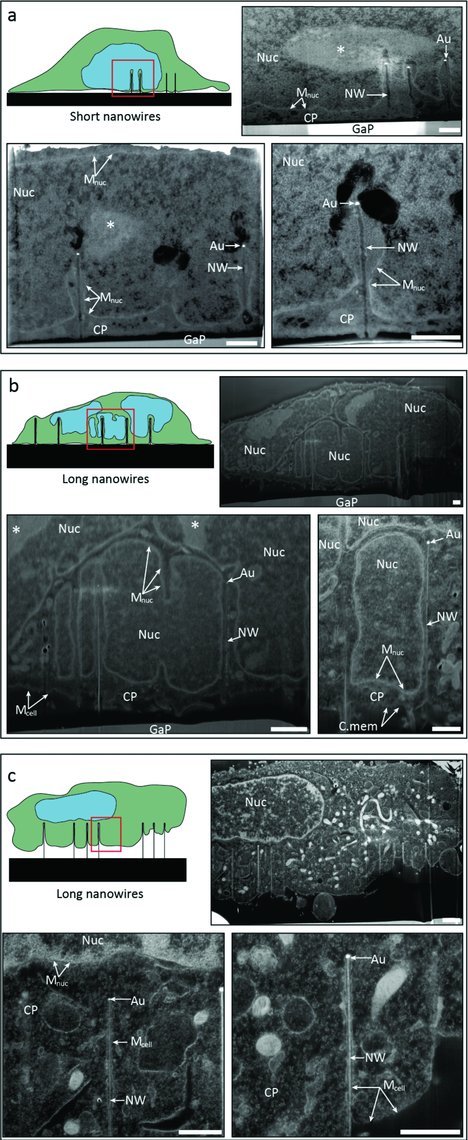
Scanning electron micrographs displaying focused ion beam milled cross sections of cells cultured for 72 h on short (a) and long (b, c) nanowires. The electron dense staining (osmium tetroxide and uranyl acetate) highlights membrane bound structures such as nuclei (Nuc), nuclear membrane (M_nuc_) and the outer cell membrane (M_cell_). The cytoplasm is labeled CP and DNA dense areas are marked with an asterisk. The gold nanoparticles used to seed nanowire growth can be clearly seen (Au), as well as the nanowires (NW). The GaP substrate is labeled GaP. Scale bars 1 μm. Images have been rescaled to compensate for tilt during acquisition.

In order to determine whether there is a direct nanowire-DNA contact, we used FIB milling and SEM to visualize the cell interior and to determine the nanowire location with respect to the nuclear membrane. **Figure** **7** shows SEM images of cell sections on short-nanowire (a) and long-nanowire (b,c) substrates. A sketch of the nanowire/cell configuration is shown for clarity. A common feature of the nanowires is that they remained vertical and appeared to penetrate the cells. On the short nanowires, the nuclear membrane remains intact (M_nuc_) and folds in order to accommodate the nanowires and exclude them from the nucleus (Figure 7a). The nuclear envelope indentation becomes extreme in the case of long nanowires (Figure 7b), where the nanowires can be seen going through tunnels or tubular membrane structures formed in the nucleus. The nanowires are clearly surrounded by nuclear membrane and excluded from the nucleus. In some cases, on long nanowires the cell does not contact the GaP substrate and no nuclear invagination can be seen (Figure 7c). In this case, the cellular membrane (M_cell_) clearly surrounds the nanowires, with the nanowire being physically excluded not only from the nucleus but from the cytosol as well. The observed enrichment of actin around the nanowires is consistent with the fact that the nanowires are surrounded by a membrane (Supporting Figure S5). A possible explanation for the variability in cell-nanowire conformation is that the local nanowire density can vary from cell to cell (since the nanowires are grown from randomly deposited aerosol gold particles on the substrate, giving only control over the average density). On an area where the density of nanowires is higher locally, cells experience a “bed of nail” effect and do not contact the base of the nanowires. This hypothesis is supported by the higher number of nanowires visible under the cell in Figure 7c compared to the cell in Figure 7b. In conclusion, the FIB-SEM images show that the nanowires do not interact directly with the DNA. Therefore other mechanisms for explaining the DNA-damage in cells must be explored.

### 2.8. Higher Content of Reactive Oxygen Species (ROS) in Fibroblasts Cultured on Nanowires

**Figure 8:**
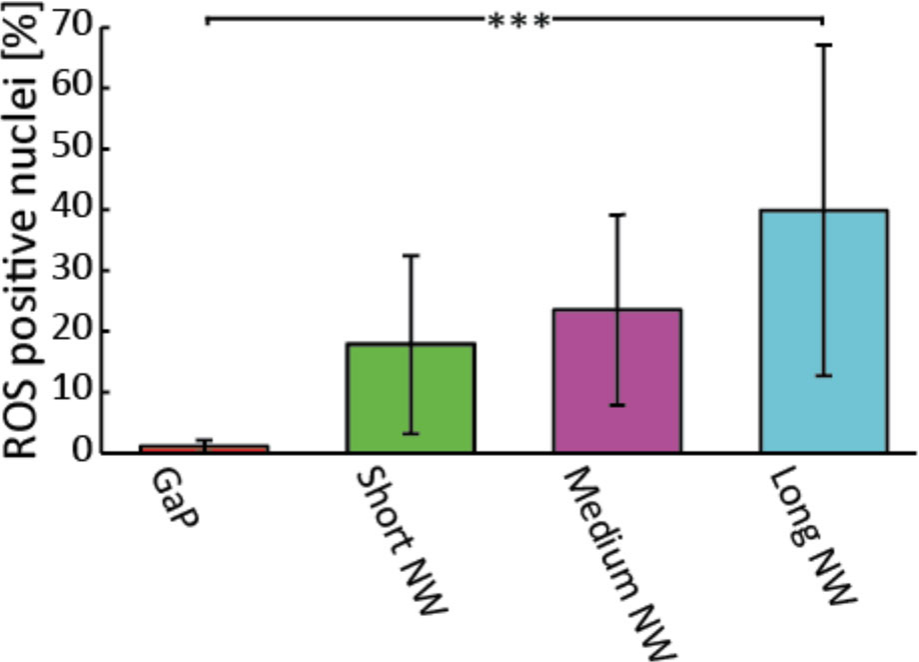
Proportion of cell nuclei positive for ROS content. Almost no control cells were positive for ROS content, while a significantly higher proportion of cells on the long nanowires were positive for ROS content. (***:p < 0.001, One way ANOVA test).

In order to examine the ROS content in cells, we used an Image-IT Live Green Reactive Oxygen Species detection kit. Our results show that fibroblasts cultured on nanowires had higher ROS content (**Figure** **8**) than those cultured on the control substrates. In particular, almost half the cells cultured on long nanowires were positive for ROS content (p < 0.001).

### 2.9. Higher Mitochondrial Metabolic Activity in Fibroblasts on Nanowires

**Figure 9:**
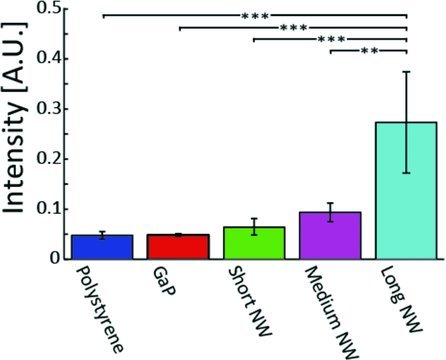
Cell respiration on nanowire and control substrates. The Alamar blue assay was used to evaluated the mitochondrial metabolic activity and showed a higher respiration rate in cells cultured on long nanowires (***: p < 0.001, **:p < 0.01).

One known source of ROS formation is cell respiration,^24^ therefore, we performed a respiratory assay on cells cultured on nanowire and control substrates. Our results show that the cells have a significantly higher respiration rate on the long nanowires (**Figure** **9**).

## 3. Discussion

Our results show that fibroblasts are less motile when cultured on nanowire substrates compared to control substrates. This confirms the fact that nanopillars can prevent cells from migrating, as recently demonstrated for neurons, for which the migration was reduced from 60 μm to 4 μm over a period of 5 days.^25^ Here, we studied fibroblasts that are highly motile compared to neurons and were able to immobilize the cells that usually migrate 200 μm in just 20 h. The SEM images indicate that the decrease in fibroblast motility and proliferation is likely caused by the nanowires physically impeding both cell movement and mitosis. When a cell is about to divide, it lifts itself from the substrate, which may be sufficient to disentangle the cell from the short nanowires but not from the long nanowires. Since the nanowires impede cell division, it is likely that this effect scales with the cell proliferation rate, which in turn leads us to the exciting possibility of using the long nanowire substrates for applications where the reduction of a proliferating cell population over a non-proliferating cell type would be beneficial (for instance neurons versus glial cells or normal cells versus cancer cells). In retinal cell cultures, we have recently observed a significant difference in the number, morphology and conformation of astroglial cells on nanowires compared to flat substrates.^2^ These proliferating cells were more spread out and had a tendency to form a confluent layer on the flat GaP substrates, while they were very few and not spread-out on the nanowire substrates. At the same time, the neuronal outgrowth was significantly enhanced by the presence of nanowires. This effect was visible on nanowires longer than 1 μm and was enhanced with increasing nanowire length. In the present paper, we show that failed cell division results in multinuclear cells on the nanowires. Although we did not detect multinuclear astroglial cells in retinal cell cultures on nanowire substrates, it is possible that the nanowires limited the proliferation and spreading of astrocytes while not affecting neurons, which are not proliferating. Another study reported the presence of multinuclear fibroblasts in cell cultures, where they were shown to be the result of, either secondary fibroblasts fusing in the presence of macrophages, or senescent primary fibroblasts no longer undergoing cytokinesis but maintaining DNA replication.^26^ However, in our case, based on the time-lapse images (Figure 4), it is more likely that the multinuclear cells we observe are the result of failed cytokinesis than the result of distinct fibroblast fusing. This raises the question whether long nanowires can drive cells into senescence, something that would be especially interesting to investigate for the case of cancer cells.

Recent studies in the literature agree on the fact that the nanowires do not spontaneously pierce the cell membrane.^4^, ^16^, ^17^ The successful transfer of biomolecules into the cytosol may instead come from the higher surface area on nanowire substrates compared to flat ones. However, other possible mechanisms cannot be excluded at this point. Our FIB–SEM results indicate that the cell nucleus is not penetrated by the nanowires. In order to avoid contact with the nanowires, the nuclear membrane folds in a complex manner. Previous studies have shown that similar invaginations and tubes can be formed in cell nuclei, both naturally and in the presence of certain drugs.^27^, ^28^ This seems to indicate that nanowires cannot be used for direct gene or drug delivery into the nucleus. Yet, gene and drug delivery has been demonstrated experimentally,^7^, ^11^ suggesting an alternative mechanisms where the high curvature of the nuclear membrane results in higher membrane permeability,^29^ thereby facilitating the transfer of molecules to the nucleus in a more effective way than by simply increasing the surface area of the substrate using nanowires.

Finally, our results show that cells on nanowires exhibit DNA damage, which cannot be explained by direct nanowire-DNA interactions. We observed a higher count of ROS positive cells on long nanowires compared to control, as well as a higher cell respiration on these substrates. We speculate that the higher rate of respiration on these substrates results in a higher number of cells positive for ROS, which in turn leads to DNA damage. The elevation in cell respiration on nanowires may be due to the fact that cells are forced to maintain membranes of higher surface area compared to cells on flat surfaces, which have a more spherical shape, both for the nucleus and cytosol. However, more experiments would be required to confirm our hypothesis as the elevated cellular respiration might arise from other forms of cellular stress.

## 4. Conclusion

We show that cells cultured on nanowire substrates have a reduced motility and proliferation rate compared to flat control substrates. Cell division is impeded by the nanowires based on the observation of several anomalies, such as a significantly higher proportion of multinuclear cells when compared to cells grown on planar substrates. Nanowire-induced DNA damage, ROS formation and increased respiration rate were also observed. Cross-sectional high-resolution imaging using FIB milling combined with SEM reveals an intact nuclear envelope not pierced by the nanowires but rather bent to an extreme extent thereby avoiding the nanowires. We therefore propose that the DNA damage is due to ROS formation induced by increased cell respiration, and not by any direct nanowire-DNA contact. These results should be taken into consideration when developing and evaluating experiments based on nanowire platforms for interaction with living cells. Specifically for device design, the nanowire length and the interaction time between cells and nanowires should be minimized in order to reduce nanowire effects on the cell behaviour. However, the effect of long nanowires on cell division and motility may be beneficial in applications where a proliferating cell population should be prevented from taking over other cells, for instance, in the case of cancer or when dealing with the immune reaction to an implant.

## 5. Experimental Section

*Sample Fabrication*: Metal organic vapour phase epitaxy (MOVPE) (Aix 200/4, Aixtron, Germany) was used to grow gallium phosphide (GaP) nanowires from 80 nm gold aerosol particles randomly distributed (average density 1 μm^−2^) on single or double-side polished (111)B GaP substrates (Girmet Ltd, Moscow, Russia), as previously described.^30^ The nanowires were grown from trimethyl-gallium and phosphine precursors. The growth time was used to control the final nanowire length and nanowires of three different lengths were fabricated: short (1.5 ± 0.1 μm), medium (3.8 ± 0.3 μm) and long (6.7 ± 0.3 μm). In this study, tissue culture plastic (polystyrene) and GaP substrates devoid of nanowires were used as controls.

*Cell Cultures for Time Lapse Imaging Experiments*: Mouse fibroblasts L929 (DSMZ, Braunschweig, Germany) were cultured in PS tissue culture flasks in RPMI1640 medium supplemented with 10% fetal calf serum, 100 U/mL penicillin and 100 μg/mL streptomycin, at 37 °C in 5% CO_2_ in water-saturated air (CO_2_ incubator). The cells were detached using trypsinization and the cell concentration was determined by counting in a haemocytometer. Nanowire and flat substrates were placed in 25 cm^2^ PS cell culture flasks (1 substrate per flask) and empty flasks were used as PS controls. All substrates were sterilized overnight using UV-radiation. Cells (approximately 200 000) were seeded in each culture flasks in 5 mL of medium and kept in a CO_2_ incubator.

For time-lapse imaging, the cells were allowed to adhere to the substrates for 24 h before being moved to the phase holographic microscope. The microscope was placed in a 37 °C dry incubator and in order to retain the 5% CO_2_ atmosphere, the culture flasks were tightly closed when removed from the standard CO_2_ incubator.

*Time Lapse Imaging*: A phase holographic microscope (HoloMonitor M3, Phase Holographic Imaging AB, Lund, Sweden),^31^ placed in an incubator, was used to capture time lapse images of cells, at 1 min intervals. Using phase holographic microscopy greatly improves the image quality of cells grown on nanowire substrates, compared to standard phase contrast microscopy (Supporting Figure S1). An important feature of the microscope is the long wavelength laser light used to image the sample (633 nm), which reduces photo-induced cell damage, allowing up to 48 h analysis periods. Finally, the fact that the method is label-free ensures a minimum impact on the cell behaviour.

*Cell Motility Analysis*: In order to quantify cell motility, the cells were tracked using the plugin program *Circadian Gene Expression* (CGE) in ImageJ (ImageJ 1.44p, National Institutes of Health, USA). For each sample, 20 cells were chosen by randomly generating 20 pairs of (X,Y)-coordinates and selecting the cell closest to those coordinates. The cells were then tracked throughout the first 20 h of the time-lapse movies and their mean square displacement was calculated.

*Growth Curves*: Cells were seeded at t = 0 h and imaged using phase holographic microscopy every 24 h for 4 days. Three replicates of each substrate (short, medium and long nanowires, as well as polystyrene and planar GaP controls) were used. Five images were captured at random locations, before the samples were returned to the CO_2_ incubator.

The number of cells in each image was then determined using the microscope software's built in cell identification tool (HoloStudio v2.4, Phase Holographic Imaging AB, Lund, Sweden).

*Long Term Growth Curves*: Fibroblasts were seeded on short nanowire, long nanowire and PS substrates (three samples of each kind). The cultures were then maintained for 360 h in a CO_2_ incubator and retrieved for imaging at t = 24 h, 96 h, 192 h, 264 h and 360 h. After imaging, the medium covering the cells was exchanged for new fresh medium (with the exception of t = 24 h). The cell density was determined according to the method described above.

*Fluorescence Labelling*: The samples used for the time-lapse imaging and growth curve experiments were fixed in 3.7% formaldehyde for 45 min and washed with PBS. The cells were stained using FITC-labelled phalloidin (Sigma-Aldrich, St. Louis, USA) at a concentration of 1.7 μg/μL in PBS with 5% dry milk (w/v) and 0.1% Tween20 (v/v) for 1 h at room temperature. The samples were washed with PBS and stained for 1 min using bisbenzimide (Hoechst 33342, Invitrogen, Carlsbad, USA) at a concentration of 1 μg/mL in PBS. The samples were subsequently washed in PBS.

Some samples were also labelled with mouse anti-β-tubulin III antibodies (Sigma-Aldrich, St. Louis, MO, USA) diluted 1:200 in PBS with 5% dry milk (w/v) and 0.1% Tween20 (v/v) for 90 min. The samples were subsequently stained with RPE-labelled goat-anti mouse antibodies (Dako, Glostrup, Denmark) at a concentration of 5 μg/μL in PBS with 5% dry milk (w/v) and 0.1% Tween20 (v/v) for 90 min. These samples were also stained using phalloidin and bisbenzimide as above.

*Confocal Microscopy*: The samples were imaged using a scanning laser confocal microscope (LSM510, Carl Zeiss SMT GmbH, Oberkochen, Germany). Low magnification, single optical plane images were used to quantify multinuclear cells on the different substrates and high magnification single optical planes were used to study multinuclear cells in detail.

*Fluorescence Microscopy*: The samples were imaged using an iXon897 camera mounted on a Nikon TE2000 microscope using standard wide field microscopy objectives.

*Focused Ion Beam Milling*: The mouse fibroblasts were cultured for 72 h on short and long nanowire substrates and fixed in 2% glutaraldehyde in 0.05 M sodium cacodylate buffer. The samples were flushed and stained with osmium tetroxide, tannic acid and uranyl acetate. After heavy metal staining the samples were dehydrated, first in a graded ethanol series and then in propylene oxide. A 1:3 Epon embedding resin to propylene oxide was applied and the propylene oxide was allowed to evaporate overnight. Samples were then cured at 60 °C for 48 h and polished to obtain a thin Epon layer above the cells (Supporting Table S3). Following a thin gold coating, the samples were imaged in a Helios Nanolab 600 dual-beam system (FEI Company, Hillsboro, USA). Using standard SEM with the highest acceleration voltage for a maximum beam penetration depth, cells of interest were located from atop using previously taken light microscopy images for guidance. Once located, rough milling at high current was performed using Ga^+^ ions (FIB) to create a trench in front of the cell. Lowering the ion beam current and electron beam voltage FIB-SEM, *slice and view* was performed to cyclically perform FIB milling and SEM imaging to image the sample and locate relevant sites with visible nanowires.

*γ-H2AX Labelling*: Cells were cultured on nanowire and control substrates placed in 24 well plates for 96 h. Cells were fixed for 20 min in 3.7% paraformaldehyde, washed with PBS and labelled for 90 min with rabbit anti- γ-H2AX (Cell Signalling Tech, Danvers, USA) (1:200) in PBS with 1% (v/v) Tween 20 and 1% (w/v) BSA (Sigma-Aldrich, St. Louis, USA) at room temperature. The samples were washed with PBS and incubated for 90 min with Alexa Fluor 594-goat-anti-rabbit (Invitrogen, Carlsbad, USA) (10 μg/mL) and FITC-labelled phalloidin (Sigma-Aldrich, St. Louis, USA) (1.7 μg/μL) in PBS with 1% (v/v) Tween-20 and 1% (w/v) BSA at room temperature. Finally the samples were washed with PBS and stained for 1 min using bisbenzimide (Hoechst 33342, Invitrogen, Carlsbad, USA) at a concentration of 1 μg/mL in PBS. The samples were then imaged with fixed imaging settings.

*Reactive Oxygen Species (ROS) Detection*: We used the Image-IT Live Green Reactive Oxygen Species detection kit (Invitrogen, Carlsbad, USA). Cells were seeded on nanowire and control substrates in 24 well plates at 8000 cells/well in Hank's buffered salt solution (HBSS) and cultured for 96 hours in an CO_2_ incubator. The cells were washed in HBSS and incubated with the detection kit dye as instructed by the manufacturer (1:400) for 30 min in the incubator before rinsing with HBSS. The cell nuclei were stained using bisbenzimide and rinsed in HBSS and imaged immediately after staining. The imaging settings were fixed for all samples.

*Cell Respiration Assay*: We investigated the cell respiration using the Alamar Blue assay (Invitrogen, Carlsbad, USA). Cells were seeded on nanowire and flat substrates in 24 well plates at 8000 cells/well and cultured for 96 hours. PS wells were used as control. Wells devoid of cells were used to quantify the background. The GaP samples were moved to new wells in 1 mL growth medium (see cell culture method above) and incubated for 1 h. Alamar blue was added to each well (10% v/v) and the plates were then incubated for 4 hours before 200 μL of medium from each well was transferred to a 96 well plate and the fluorescence was measured in a spectrophotometer using 530 excitation/590 emission filter settings. The cell nuclei were stained using bisbenzimide and the total number of nuclei was evaluated on each substrate. The cellular respiration was normalized to the number of cell nuclei on the substrate.

## Supporting Information

Movie001

Movie002

Movie003

Movie004

Movie005

Movie006

Suppl
