# Supplementary material for: Fibroblasts Cultured on Nanowires Exhibit Low Motility, Impaired Cell Division, and DNA Damage
Source: Small. 2013 Jun 27;9(23):4006–16. doi: 10.1002/smll.201300644 (PMC4282547; doi:10.1002/smll.201300644)
Supplement: Supplementary file 7 — Suppl [file smll0009-4006-SD7.pdf]

Copyright WILEY-VCH Verlag GmbH & Co. KGaA, 69469 Weinheim, Germany, 2013.

NANO small MICRO

Supporting Information

for *Small*, DOI: 10.1002/smll.201300644

Fibroblasts Cultured on Nanowires Exhibit Low Motility, Impaired Cell Division, and DNA Damage

*Henrik Persson, Carsten Købler, Kristian Mølhave, Lars Samuelson, Jonas O. Tegenfeldt, Stina Oredsson, and Christelle N. Prinz**

Supporting information for

Fibroblasts Cultured on Nanowires Exhibit Low Motility, Impaired Cell Division and DNA Damage.

Henrik Persson, Carsten Købler, Kristian Mølhave, Lars Samuelson, Jonas O. Tegenfeldt, Stina Oredsson and Christelle N. Prinz*

Henrik Persson, Prof. Lars Samuelson, Dr. Jonas O. Tegenfeldt

Division of Solid State Physics/The Nanometer Structure Consortium, Lund University, Box 118, 22100 Lund, Sweden.

Carsten Købler

Center for Electron Nanoscopy and Department of Micro- and Nanotechnology, Technical University of Denmark, , Ørsteds Plads 345E, 2800 Kongens Lyngby, Denmark.

Dr. Kristian Mølhave

Department of Micro- and Nanotechnology, Technical University of Denmark, Ørsteds Plads 345E, 2800 Kongens Lyngby, Denmark.

Prof. Stina Oredsson

Department of Biology/The Nanometer Structure Consortium, Lund University, Sölvegatan 37, 223 62 Lund, Sweden.

* Dr. Christelle N. Prinz

Neuronano Research Center and Division of Solid State Physics/The Nanometer Structure Consortium, Lund University, Box 118, 22100 Lund, Sweden.

Email address: christelle.prinz@ftf.lth.se

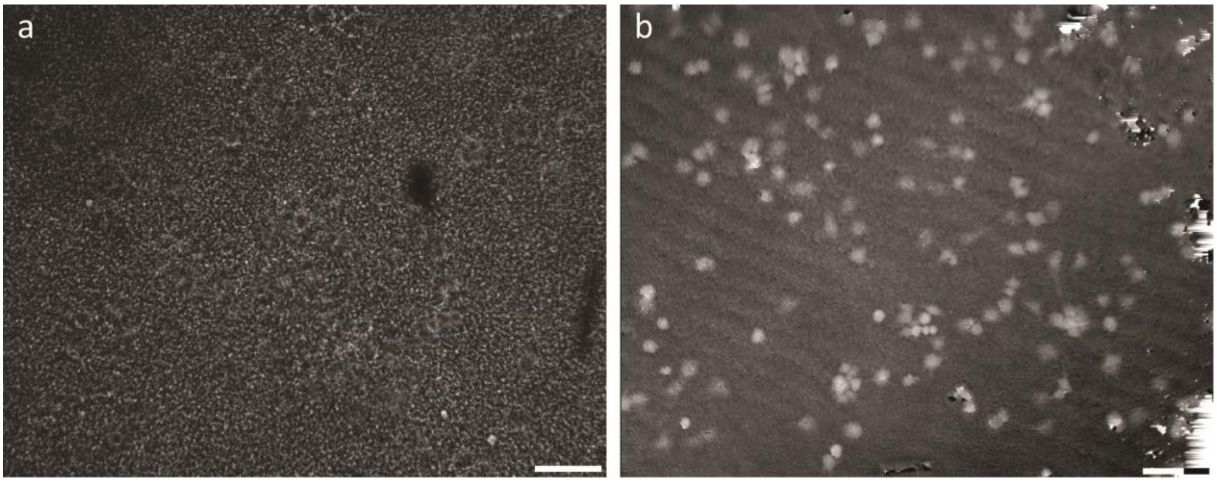

Figure S1: Comparison of images of L929 mouse fibroblasts cultured for 24 h on long nanowires. (a): image captured using standard phase contrast microscopy. (b): image captured using phase holographic microscopy. Scale bars 100 μm.

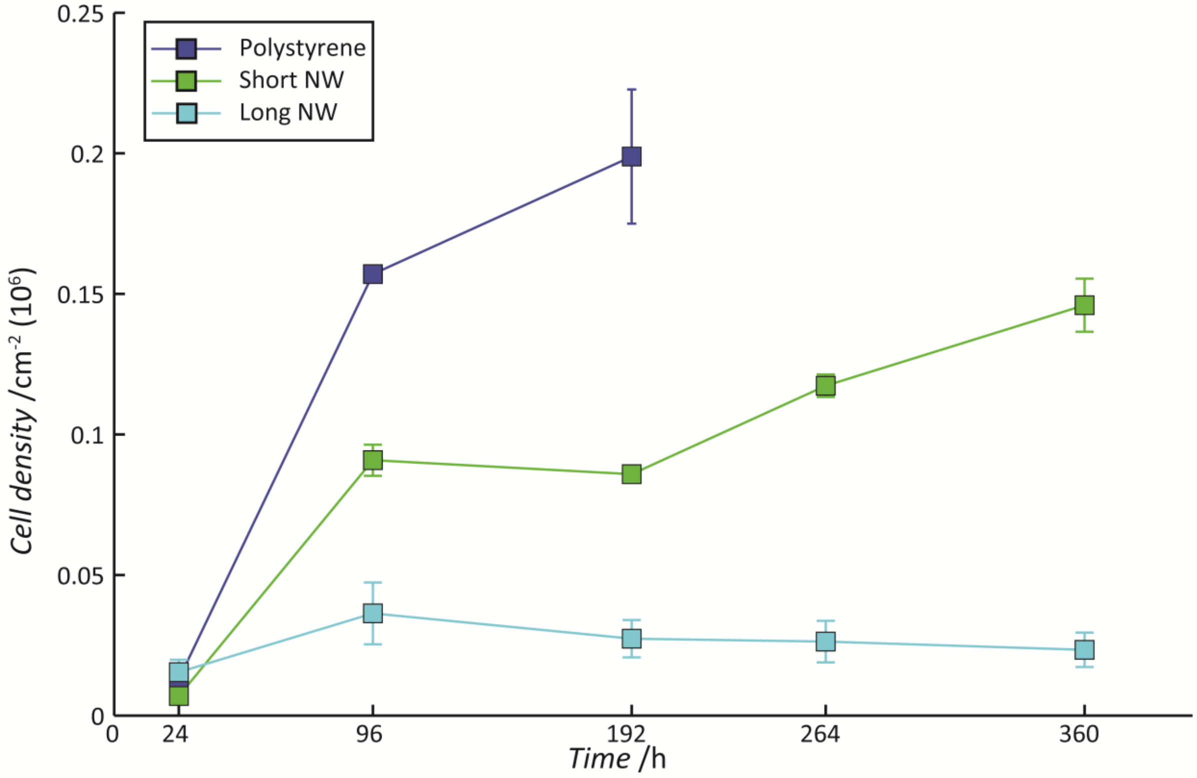

Figure S2 Growth curves of L929 fibroblasts cultured for 360h on short and long nanowires (NW) as well as on polystyrene. Data generated using holographic microscopy (n=3). Mean value ±S.E.M.

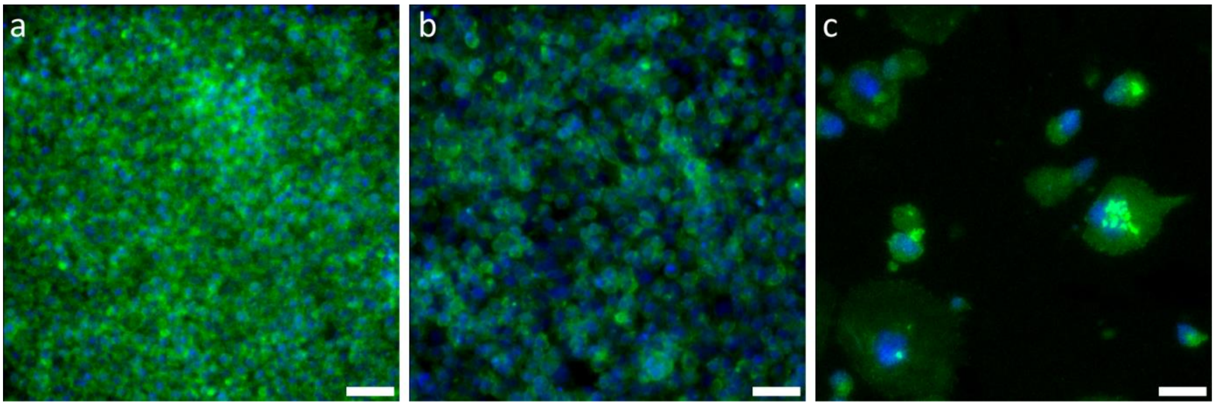

Figure S3: Fluorescence overview images of L929 mouse fibroblasts cultured for 360 h on polystyrene (a), short nanowires (b) and long nanowires (c). There is a clear difference in cell density between the different samples. Cells on polystyrene are very dense and the cells appear to grow on top of each other. Cells on short nanowires are confluent but the cells on long nanowires still have a very low density. The cells on long nanowire substrates also have a strikingly different morphology, with very large cells, often with multiple and/or large and deformed nuclei (Fig. S4). Actin has been labeled with FITC-conjugated phalloidin (green) and the nuclei have been labeled with bisbenzimide (blue). Scale bars 50 µm.

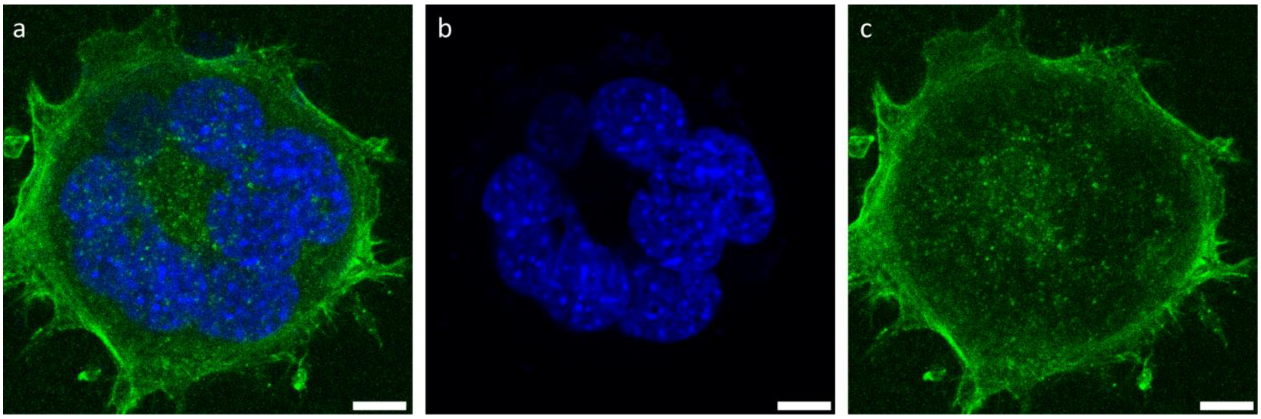

Figure S4: Confocal microscopy image of a single L929 mouse fibroblast with 9 cell nuclei cultured on long nanowires for 360 h. The actin cytoskeleton is stained with FITC conjugated phalloidin (green) and the nuclei are labeled with bisbenzimide (blue). Scale bars 10 µm.

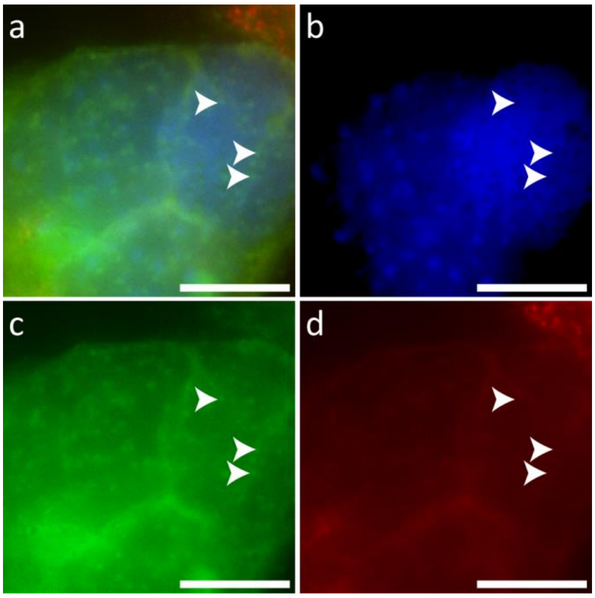

Figure S5: Fluorescence microscopy image of a tri-nuclear cell cultured for 96 h on a long-nanowire substrate. The actin filaments were stained with FITC-labeled phalloidin (green), the nuclei were labeled with bisbenzimide (blue). The microtubules were labeled with mouse antibodies against β-tubulin and RPE-labeled goat anti-mouse antibodies (red). Dark dots are visible in the nucleus (arrows), which suggests that the nucleus is pieced by the nanowires. These dark spots are co-localized with actin (green dots) but not with microtubules. Scale bars 10 µm.

Sample	Dividing cells	Multinuclear cells	Cell density
**Polystyrene**	2.0±0.2%	0.1±0.1%	200,000±2,000cm^{-2}
**Plain GaP**	1.1±0.8%	1±1%	119,000±14,000 cm^{-2}
**Short NW**	0.6±0.2%	0.4±0.2%	116,000±1,500 cm^{-2}
**Medium NW**	0±0%	3.7±1.0%	70,000±2,500 cm^{-2}
**Long NW**	0±0%	16±5%	27,000±900 cm^{-2}

Table S1: Percentage of large, multinuclear L929 cells on the different samples after 72 h of incubation

Sample	Dividing cells	Multinuclear cells	Cell density
**Polystyrene**	-	-	590’000cm^{-2}
**Short NW**	0	2±1.5%	230’000 ± 90000cm^{-2}
**Long NW**	0	31±8%	18’000 ± 7’000cm^{-2}

Table S2 Percentage of large, multinuclear L929 cells on the different samples after 360 h of incubation. Due to the high cell density, it was not possible to count cells on the polystyrene, thus the proportion of multinuclear cells could not be determined.

Step	Chemicals	Time
**Fixation**	2% GDA (0.2 M) + 0.05 M cacodylate buffer (total 300 mOsm)	Min. 1 h
**Flush**	0.15 M cacodylate buffer	2 X 30 min
**Post-fix/stain**	1 % OsO_4 + 0.12 M cacodylate buffer	1 h
**Rinse**	Milli-Q water	2 X 10 min
**Mordant**	1%wt tannic acid in Milli-Q water	1 h
**Rinse**	Milli-Q water	2 X 10 min
**Stain**	1%w/w Uranyl acetate in Milli-Q water	2 h (can be left overnight)
**Dehydration**	70% EtOH	2 X 10 min ethanol (can be stored in 70% EtOH)
**Dehydration**	96% EtOH	2 X 10 min
**Dehydration**	100% EtOH	2 X 10 min
**Dehydration**	Propylene oxide	2 X 10 min
**Embedding**	1:3 Epon / Propylene oxide	overnight
**Curing**	Cure at 60 deg.	48 h

Table S3 Protocol for fixating, staining and embedding cells prior to FIB milling and SEM imaging.

Information on movies S1-S6

The movies were obtained using phase holographic microscopy. The color-scale to the left of the image can be used to determine the thickness of the cells on the substrate.

- Movie S1: L929 mouse fibroblasts cultured on polystyrene.
- Movie S2: L929 mouse fibroblasts cultured on plain GaP
- Movie S3: L929 mouse fibroblasts cultured on short nanowires.
- Movie S4: L929 mouse fibroblasts cultured on medium nanowires.
- Movie S5: L929 mouse fibroblasts cultured on long nanowires.
- Movie S6: L929 mouse fibroblasts cultured on long nanowires. Close-up of cell division.
